# Feasibility of screening for critical congenital heart disease using pulse oximetry in Indonesia

**DOI:** 10.1186/s12887-022-03404-0

**Published:** 2022-06-27

**Authors:** Indah K. Murni, Tunjung Wibowo, Nadya Arafuri, Vicka Oktaria, Lucia K. Dinarti, Dicky Panditatwa, Linda Patmasari, Noormanto Noormanto, Sasmito Nugroho

**Affiliations:** 1 Department of Child Health, Dr. Sardjito Hospital/Faculty of Medicine, Public Health and Nursing, Universitas Gadjah Mada, Yogyakarta, Indonesia; 2 Center for Child Health-Pediatric Research Office, Faculty of Medicine, Public Health and Nursing, Universitas Gadjah Mada, Yogyakarta, Indonesia; 3 Department of Biostatistics, Epidemiology and Public Health, Faculty of Medicine, Public Health and Nursing, Universitas Gadjah Mada, Yogyakarta, Indonesia; 4 Department of Cardiology, Faculty of Medicine, Public Health and Nursing, Universitas Gadjah Mada, Dr. Sardjito Hospital, Yogyakarta, Indonesia

**Keywords:** Congenital heart disease, Pulse oximetry screening, Indonesia, Critical congenital heart disease

## Abstract

**Background:**

Screening of critical congenital heart disease (CCHD) using pulse oximetry is a routine procedure in many countries, but not in Indonesia. This study aimed to evaluate the feasibility of implementing CCHD screening with pulse oximetry for newborns in Yogyakarta, Indonesia.

**Methods:**

A cross-sectional study was conducted at four hospitals in Yogyakarta, Indonesia. Newborns aged 24–48 hours who met the inclusion criteria were screened on the right hand and left or right foot using a pulse oximeter. Positive results were indicated by: either (1) SpO_2_ level < 90% in one extremity, (2) SpO_2_ level of 90–94% in both right hand and either foot on three measurements conducted 1 hour apart, or (3) a saturation difference > 3% between the upper and lower extremity on three measurements conducted 1 hour apart. Positive findings were confirmed by echocardiography.

**Results:**

Of 1452 newborns eligible for screening, 10 had positive results and were referred for echocardiographic evaluation. Of those, 8 (6 per 1000 live birth, 8/1452) had CCHD. Barriers found during screening processes were associated with hospital procedures, equipment, healthcare personnel, and condition of the newborn.

**Conclusion:**

Pulse oximetry screening might be feasible to be implemented within the routine newborn care setting for CCHD in Indonesia. In order to successfully implement pulse oximetry screening to identify CCHD in Indonesia, the barriers will need to be addressed.

**Supplementary Information:**

The online version contains supplementary material available at 10.1186/s12887-022-03404-0.

## Background

Congenital heart disease (CHD) is the most common congenital abnormality in newborns [1] with a reported incidence of 4 to 50 per 1000 live births [2, 3]. Approximately 25% of CHD are classified as critical congenital heart disease (CCHD), that are often lethal and require immediate transcatheter or surgical intervention in the first year of life [4]. Furthermore, CHD is responsible for over 260,000 deaths annually worldwide [5] with a CCHD associated mortality count of 34.8% in developing countries [6]. Challenges primarily exist in early detection of CCHD, with some CCHD newborns prematurely sent home before diagnosis, since they may appear healthy at first. This challenge is considerably noticeable in resource limited settings.

In Indonesia, approximately 2.5 per 1000 live births suffer from CHD [7]. A significant delay in CHD diagnosis is seen in 6 out of 10 cases, most with severe complications [8]. Additionally, one-third of the newborns with CCHD were not detected before discharge [9]. Pulse oximetry screening for CCHD has been recommended and widely implemented in many countries, leading to a significant reduction in mortality among newborns with CCHD. Furthermore, unnecessary costs related to complications due to late diagnosis of CCHD can be avoided [10]. Studies on the feasibility of pulse oximetry screening to detect CCHD have been conducted in low- and middle-income country setting including South Africa [11], India [12], Sri Lanka [13] and Brazil [14]. Despite the importance shown in the immediate detection of CCHD, no screening program has been implemented in Indonesia, contributing to the often presentation of late and even terminal cases at tertiary hospitals. Therefore, this study aimed to evaluate the feasibility of CCHD screening using pulse oximetry and provide relatable evidence for local and national policymakers in implementing pulse oximetry screening program in Indonesia.

## Methods

A cross-sectional study was conducted at four hospitals in Yogyakarta, Indonesia from August 1st, 2021, to November 30th, 2021. The hospitals were: Dr. Sardjito a class A, tertiary referral hospital; JIH a class B, general hospital; and Sadewa and Sakina Idaman, both class Cs maternal and neonatal care specialty hospitals. All seemingly healthy newborns were included, and those born at < 35 weeks’ gestation age, prenatally diagnosed with CHD, carrying dysmorphic features or signs of cardiovascular abnormalities such as cyanosis, cardiac murmur or those with abnormal vital signs were excluded [15, 16].

**Fig. 1:**
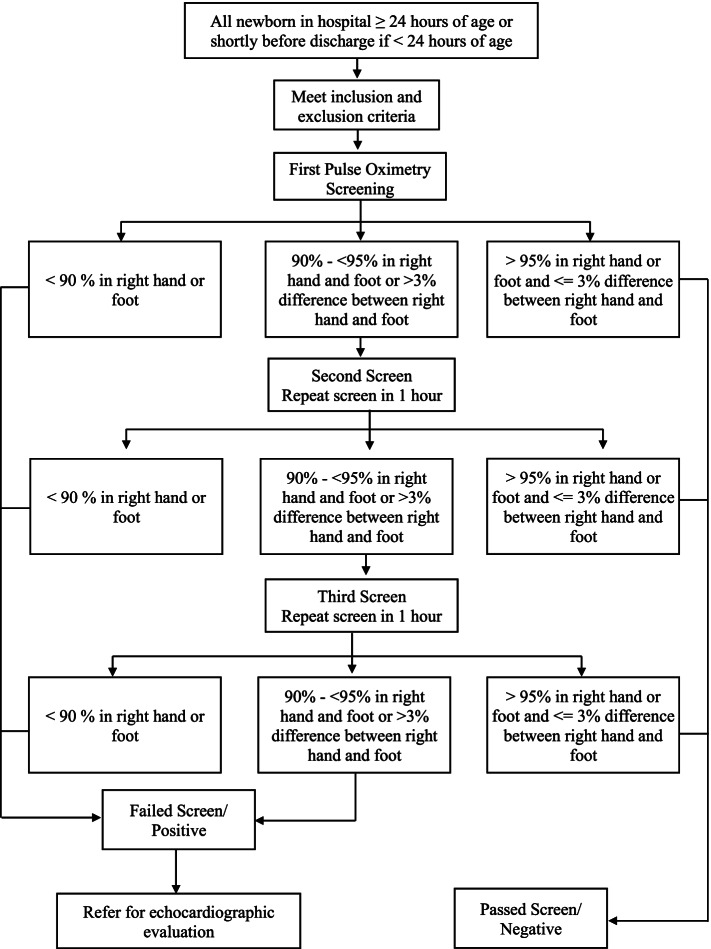
Algorithm of study protocol (Adapted from the protocol in Ewer et al. [17])

Pulse oximetry screening was performed using the American Academy of Pediatrics (AAP) standardized algorithm by measuring oxygen saturation of the right hand and the left or right foot between 24 and 48 hours of age or before 24 hours of age if the baby is discharged early. Screening of CCHD was considered negative or passed if measurement of SpO_2_ was > 95% for both the right hand and right or left foot, with a difference of < 3% between the right hand and either foot. No further cardiac evaluation was performed in these subjects unless indicated by subsequent clinical condition(s). Screening was considered positive or failed if at least one of the following: (1) SpO_2_ level < 90% in one extremity, (2) SpO_2_ level of 90–94% in both right hand and either foot on three measurements conducted 1 hour apart, or (3) a saturation difference > 3% between the upper and lower extremity on three measurements conducted 1 hour apart [17]. Subjects failing the screening were referred to Dr. Sardjito Hospital for echocardiographic evaluation. The algorithm of study is presented in Fig. 1.

Screening was performed by a healthcare worker, which included either a doctor, nurse, or midwife in charge. The measurement was written manually in case report form. Training of healthcare workers was conducted prior to the study to avoid variability in screening procedures. To compensate for the variability of available oximeter types among hospitals, we performed an agreement correlation before recruitment using three types of pulse oximeters: Massimo (Massimo Corporation, Irvine, CA, USA), Mindray (Mindray Cooperation, Nanshan, Shenzhen, China) and fingertip.

Data were analyzed using STATA version 12.1 (StataCorp, College Station, Texas, USA) and presented appropriately. Descriptive statistics were presented as numbers and percentages, mean or medians.

A semi-structured interview on barriers experienced by the medical personnel throughout the screening process was also conducted. The qualitative data were then reviewed, defined and presented thematically based on the common barriers.

### Ethics

The Medical and Health Research Ethics Committee, of the Faculty of Medicine, Public Health, and Nursing, Universitas Gadjah Mada, Yogyakarta, Indonesia has approved this study (230/UN.1/FKKMK.3/IKA.2/TU/PT.01.04/2021). Informed consent to participate in the study was obtained from the parents or legal guardians of participants.

All experiment protocols involving humans were in accordance with national/international/institutional guidelines or the Declaration of Helsinki.

## Results

**Fig. 2:**
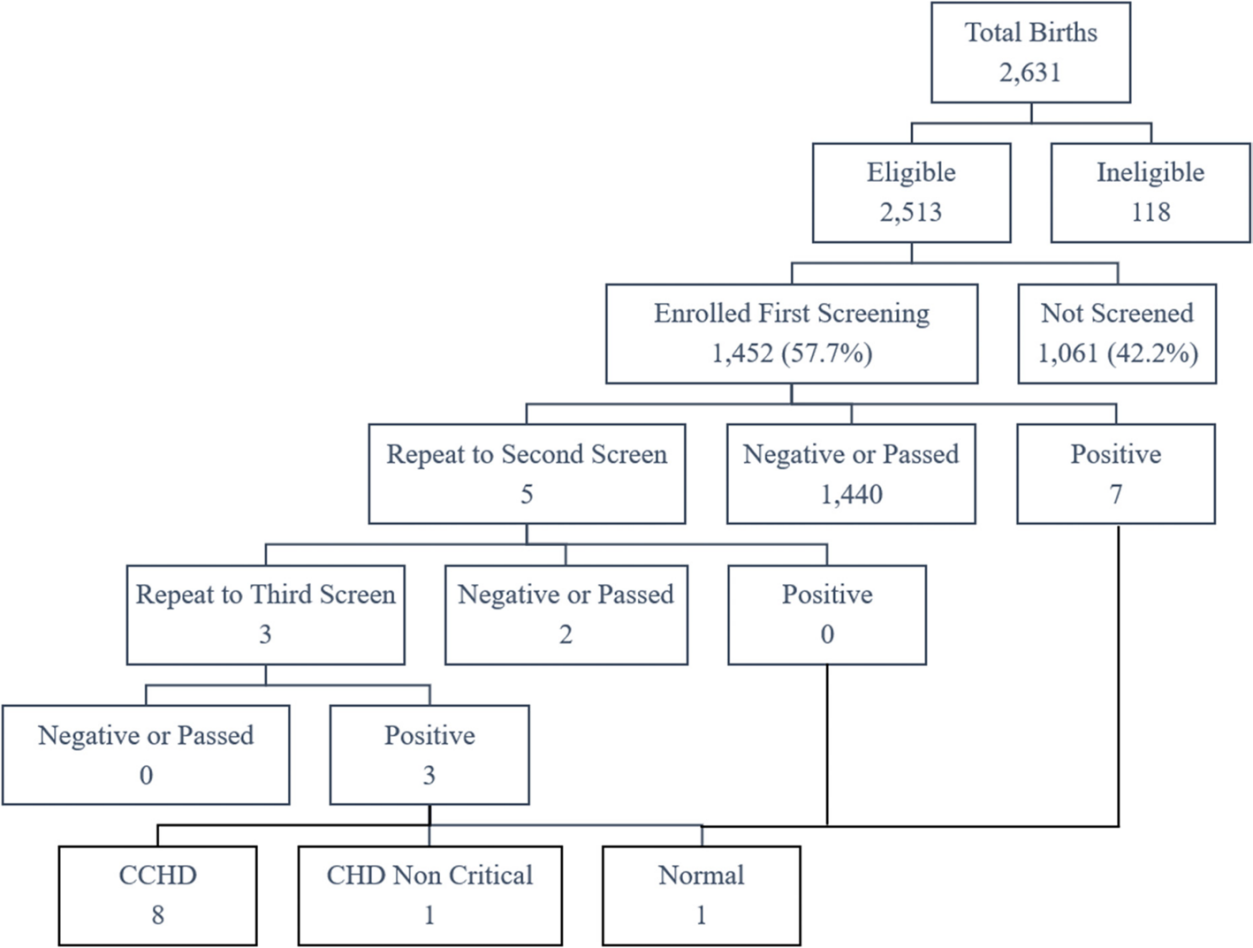
Distribution of the newborns enrolled in the study

Throughout the study period, there were 2631 newborns delivered at the four selected hospitals. From 118 newborns who were ineligible, 89 were < 35-weeks’ gestation age, 10 passed away, 14 were prenatally confirmed with CHD and 5 had dysmorphic features. A total of 1452 (57.7%) from the remaining 2513 eligible newborns were then screened (Fig. 2). Of the 1452 babies screened, 7 babies had positive results at the first screening and only 5 (0.3%) needed a second screening. Two of the babies passed the second screening. The third screening results were positive for all of the remaining three babies. Of those, 10 had positive results and were referred for further echocardiography confirmation, finally resulting in 8 (0.6%) subjects with CCHD. The screening was performed within a period of ≤24 hours after birth in 855 subjects (59%) and after 24 hours in 597 (41%) subjects.

Oxygen supplementation was promptly administered in cases where newborns were visibly bluish or when desaturation of SpO_2_ levels or signs of respiratory distress were apparent. These frequently occurred soon after birth (less than 24 hours) and was most commonly caused by asphyxia, pulmonary hypertension of the newborn, or other pulmonary problems. The healthcare workers did not include these neonates for screening.

Most screening was performed using the standardized pulse oximetry (Massimo). The agreement among Massimo and Fingertip pulse oximetry was 0.815, while the agreement among Massimo and Mindray pulse oximetry was 0.943.

The baseline characteristics of the eligible newborns are presented in Table 1. Echocardiography results performed in the 10 newborns with positive screening are shown in the Table 2.

**Table 1:** Baseline characteristics of screened newborns

Characteristics	Newborns *n* = 1452 (%)
Sex, n (%)	
Male	769 (53)
Female	683 (47)
Birth weight in gram, median (min-max)	3045.4 (1360-4532)
< 2500	154 (10.6)
2500–4000	1279 (88.1)
> 4000	19 (1.3)
Gestational age in weeks, n (%)	
35- < 37	81 (5.6)
37–42	1367 (94.1)
> 42	4 (0.3)
Type of delivery, n (%)	
Caesarean section	859 (59.2)
Vacuum extraction	29 (2)
Normal	564 (38.8)
Type of pulse oximetry, n (%)	
Massimo	1067 (73.5)
Fingertip	18 (1.2)
Mindray	367 (25.3)

**Table 2:** Echocardiography results of 10 newborns with positive screening by pulse oximetry

CCHD (*n* = 8)	Non CCHD (*n* = 2)
2 cases of Ebstein anomaly	1 case of small secundum ASD
1 case of pulmonary atresia with ventricle septal defect (VSD) and vertical patent ductus arteriosus (PDA);	1 case of patent foramen ovale (PFO) (considered normal)
1 case of tricuspid atresia with pulmonary atresia, small secundum atrial septal defect (ASD)	
1 case of mitral atresia with transposition of the great arteries (TGA), severe pulmonary stenosis, and single ventricle with hypoplastic left ventricle	
1 case of tricuspid atresia, inlet VSD, moderate secundum ASD, small right ventricle and pulmonary stenosis	
1 case of double outlet right ventricle (DORV) with TGA, VSD	
1 case of unbalanced atrioventricular septal defect (AVSD) with moderate PDA.	

Main barriers during process of pulse oximetry screening are shown in Table 3.

**Table 3:** Barriers during screening process

Type of barriers	Details
Hospital procedure	The standard hospital procedure for the length of postnatal stay is relatively short, and therefore, most of the newborns were screened before 24 hours.
	Pulse oximetry measurement has yet to be part of the pre-discharge standard care for healthy newborns, and therefore, several healthcare personnel (especially nurses and midwives) did not routinely conduct measurements despite the ongoing study.
	Among subjects with positive screening results, echocardiography examinations were not all immediately performed. This was mainly caused by the availability of echocardiographs only at the tertiary and general hospitals, while some subjects were inpatients at the other two hospitals.
Equipment	The lack of pulse oximetry devices in the common wards, with devices only available at the neonatal ICU.
	Tightly fixed sensors using Velcro or rubber fasteners were not widely available, despite being easier and faster to use compared to fingertip-type pulse oximetry.
	Adult probes were sometimes utilized due to the limited resources in the ward.
Healthcare personnel	Healthcare personnel were often occupied with other clinical duties causing them to forget to perform the screening.
Condition of the baby	Some newborns were constantly crying or moving, making measurement of SpO_2_ difficult to perform using pulse oximetry.

## Discussion

This study explored the feasibility of implementing CCHD screening with pulse oximetry for 1452 newborns in Yogyakarta, Indonesia. The results of the study indicate that pulse oximetry screening might be feasible to be implemented within the routine newborn care setting for CCHD in Indonesia.

The prevalence of CCHD in our study was 6 of 1000 live births, with positive CCHD screens occurring in 8/1452 (0.6%) of newborns. This was higher than previously reported in Sri Lanka 0.16% (14/8718), [13], India 0.16% (3/1855) [12], Turkey 0.12% (12/10,200) [18], Morocco 0.06% (5/8013) [19], the Netherlands 0.02% (5/23,959) [20], New Zealand 0.02% (3/16,644) [21], South Africa 0.01% (1/1001) [11] and the United States (US) 0.01% (1/6745) [16].

A meta-analysis of 21 studies involving 457,202 participants concluded that pulse oximetry is a highly specific and moderately sensitive test for detection of CCHD with very low false-positive rates [22]. Pulse oximetry screening has been successfully implemented in high-income countries and has led to a significant reduction in CCHD related deaths. A study showing results from a 6 years evaluation (2007–2013) after implementation of pulse oximetry screenings across the United States found a 33.4% (95% CI: 10.6–50.3%) reduction in CCHD deaths per 100,000 births, with a further potential reduction of 120 infant deaths per year from CCHD [23].

Several countries that have already conducted CCHD screening programs with pulse oximetry such as the US, China, the Netherlands, and the United Kingdom have indicated the practice combined with clinical assessment is beneficial and cost-effective [10]. Through the program, costs for treating complications due to the late diagnosis of CCHD can be avoided. A US study reported the screening program saves 20 infants annually, with an equivalent cost of $40,385 per life-year gained under base case assumptions that each screening would cost $6.28 per newborn [24].

Meanwhile, low- and middle-income countries still must face several barriers to be able to execute the pulse oximetry screening program. One study in Morocco revealed barriers such as the tendency to discharge healthy newborns before 24 hours, and the difficulty in confirming positive screening results due to the lack of available echocardiographs in several hospitals [19]. Other reported challenges in implementing pulse oximetry screening include acceptance of the program, timing of screening and significance of false positives rate, and response to positive screen results [25].

In our study, we classified barriers found during screening into four concerns. The first concerns involve hospital procedures or workflow. The AAP recommends screenings should be done within 24–48 hours of age. Adversely, most subjects in our study were screened before 24 hours due to the relatively short postnatal length of stay for healthy babies decided in hospital procedures. The timing of screening should be considered since it will influence the screening results. A previous study revealed that the measurement of saturation before 24 hours of age will increase the false positive or false negative rate [20]. The transition from fetal to neonatal circulation and stabilization of systemic oxygen saturation levels might explain this finding. A New Zealand study revealed that a midwifery-led maternity setting characterized by early discharge, influenced the time of testing, effecting saturation levels [21].

The second barrier involves the scarcity of standardized neonatal pulse oximeters, with devices readily available in neonatal intensive care units or observation rooms for monitoring sick newborns, but not in postnatal wards. Only some pulse oximeters were equipped with tightly fixed sensors using Velcro or rubber fasteners which are easier to use compared to finger-type devices where the pulses tend to be difficult to detect and take longer to read. Owing to limited sources, some hospitals even resorted to using adult probes for newborns. The type of probe can affect the effectiveness of examinations. The US Food and Drugs Administrator (FDA) stated three recommendations in using a pulse oximeter: (1) be aware to the factors that can affect the accuracy of a pulse oximeter reading, (2) understand the particular brand and sensor by referring to the device labelling or manufacture’s website, and (3) always consider accuracy limitations when using a pulse oximeter to assist in diagnosis and treatment. Knowing these recommendations is important in understanding the risk of measurement inaccuracy and providing the highest outcomes [26]. Nevertheless, a recent study revealed that a pulse oximetry device provided good accuracy in ruling out hypoxemia in comparison to saturation reading by arterial blood gas sample [27].

The third barrier is related with the condition of the baby. During our study, some newborns were constantly crying or moving, posing a challenge in the application and assessment of pulse oximetry. It is recommended that infants should be fully awake but settled during the screening process, since deep sleep may result in hypoventilation and low saturation results [28]. A previous study in New Zealand showed that newborns that were asleep or unsettled during screening were less likely to have positive results than those who were awake but settled [21].

The fourth and last of the barriers is the lack of healthcare personnel in the postnatal ward. The healthcare providers were occupied with other clinical duties and sometimes forgot the screening protocol. Some did not consider the pulse oximetry measurement to be within the scope of their practice due to low motivation because no incentive was given. A study in South Africa reported that most of the nurses involved in the study were satisfied with the purpose and aim of the study, but they do not have enough time to do the screening since their workloads were already heavy [11]. A study in New Zealand stated that most of midwives agreed that pulse oximetry screening was beneficial, but their already heavy workload prevented them from routinely performing screens. This was one of their concerns regarding the implementation of pulse oximetry as a universal screening program [29].

Indonesia has a large annual live birth rate, at 5 million per year with around 62.7% deliveries commonly assisted by midwives. As many as 79% of women gave birth at health care centers, with around 16% giving birth at home [30]. Nevertheless, Indonesia still lacks any national program for CCHD screening [8]. Pulse oximetry fulfils the criteria for mass screening. It is very effective, low cost and can significantly reduce morbidities and mortality by providing earlier detection of CHD. However, to achieve these goals optimally in a setting where resources are limited is challenging, though not impossible. These goals require extensive standardized training for healthcare providers who work directly in childbirth and newborn care (midwives, nurse, and general practitioner), the measurement protocols need formal regulations and the involvement of policy makers such as health ministries and the pediatric cardiology society to make pulse oximetry screening a recommendation in the standard care of newborns. Further, in order to develop an appropriate system for home birth, the timing of administration of pulse oximetry might need to be altered since a community midwife leaves approximately several hours after an uncomplicated home birth. Extensive training for community midwives and providing each midwife with a handheld pulse oximeter also need to be conducted. However, in order to make this approach works, an appropriate regional system to support the use of pulse oximetry in individual home births should be developed.

In order to optimize the impact of pulse oximetry screening in low-middle income countries, Zheleva et al. summarized several recommendations to be considered including the assessment of referral CHD health services, assessment of birth delivery center processes and staff training needs, financial burden and implementation of CCHD screening process as part of the overall patient care continuum [31]. In principle and practice, pulse oximetry screening is relatively simple, inexpensive, and easy to implement. However, screening is just one step in a lifelong continuum of management for the child diagnosed with CCHD and their family. If a child has a positive screening for CCHD, they require immediate access to definitive diagnosis, safe transportation, and surgical and interventional cardiology services. Newborn screening cannot save as many lives as it should if high quality cardiac services are not available to the child and family following a positive screen. It will help detect cases, but many will not survive or will live a life with serious disability.

The major limitations of our study involve the high proportion of newborns who were not screened over the study period due to the many aforementioned reasons and there was no report provided on parent’s acceptance of and uptake of the pulse oximetry screening for CCHD. This study was also limited by the use of three different types of pulse oximeters from three different manufacturers as well as the use of an adult sensor. Despite the limitations, our study is among the first reports of the feasibility of CCHD screening using pulse oximetry in Indonesia and provides the local evidence of barrier perspectives from healthcare workers during the screening process. The findings can be used as available local evidence for policymakers before recommending needed changes to the national screening program.

## Conclusions

Pulse oximetry screening might be feasible to be implemented within the routine newborn care for detection of CCHD in Indonesia. In order to successfully implement pulse oximetry screening to identify CCHD in Indonesia, the barriers will need to be addressed.

## Supplementary Information

**Additional file 1.**

## Data Availability

All data generated or analyzed during this study are included in this published article [and its supplementary information files].

## Abbreviations

AAP: American Academy of Pediatric
ASD: Atrial Septal Defect
CHD: Congenital Heart Disease
CCHD: Critical Congenital Heart Disease
DORV: Double Outlet Right Ventricle
PDA: Patent Ductus Arteriosus
PFO: Patent Foramen Ovale
TGA: Transposition of the Great Aorta
VSD: Ventricle Septal Defect
